# Primitive neuroectodermal tumor originating in the vulva: A case report

**DOI:** 10.3892/ol.2014.2073

**Published:** 2014-04-16

**Authors:** MAKIKO MATSUDA, TOMOYUKI ICHIMURA, MARI KASAI, MAKOTO MURAKAMI, MANABU HOSHI, NAOKI KAWAMURA, TOSHIYUKI SUMI

**Affiliations:** 1 Department of Obstetrics and Gynecology, Osaka City University Graduate School of Medicine, Osaka 545-8585, Japan; 2 Department of Obstetrics and Gynecology, Osaka City Sumiyoshi Hospital, Osaka 559-0012, Japan; 3 Department of Orthopedic Surgery, Osaka City University Graduate School of Medicine, Osaka 545-8585, Japan; 4 Department of Obstetrics and Gynecology, Osaka City General Hospital, Osaka 534-0021, Japan

**Keywords:** primitive neuroectodermal tumor, vulva, MIC-2

## Abstract

Primitive neuroectodermal tumors (PNETs) exhibit chromosomal translocations in common with those of Ewing’s sarcoma. They usually originate in bone or soft tissue but rarely arise in the vulva. The current case report presents a case of PNET originating in the vulva in a 60-year-old female, who previously underwent enucleation of a vulvar tumor in another hospital. The pathologist suspected a histopathological diagnosis of PNET, and simple vulvectomy and resection of the inguinal lymph nodes were performed. An ~3 cm mass recurred in the right side of the vulva four years following the initial surgery and the tumor was excised. The tumor comprised small, round-to-oval nuclei and stained positively for MIC-2, synaptophysin, neuron-specific enolase and neurofilament antibodies. To date, the patient remains alive and with no evidence of disease four years following multidisciplinary treatment, despite PNETs usually exhibiting a poor prognosis. This is due to the small tumor size and the absence of distant metastasis.

## Introduction

Primitive neuroectodermal tumors (PNETs) were first discovered by Hart and Earle in 1973 (1). PNETs are small, round cell tumors that undergo neuroectodermal differentiation. PNETs exhibit chromosomal translocations in common with those of Ewing’s sarcoma (EWS) and are thus often called Ewing family tumors (EFTs). PNETs are classified as central or peripheral types based on their site of origin, they usually originate in bone or soft tissue, but rarely arise in the vulva. A diagnosis normally based on morphological features and the immunohistochemical staining profile in addition to cytogenesis and clinical symptoms. Typical histological features include small, uniform sized round cells with hyperchromatic nuclei and scant cytoplasms. The tumors exhibit neural differentiation by forming a rosette-like structure. However, they are normally diagnosed by immunohistochemical methods. In the vast majority of cases, PNETs have been shown to express at extremely high levels, an antigen determined by the MIC2 gene. PNET patients usually experience pain as the tumors may develop in almost any bone or soft tissue. Approximately 25% of patients have detectable metastatic lesions in the lung, bone and bone marrow at diagnosis. The current case report presents a case of PNET originating in the vulva.

## Case report

### Patient presentation

A 60-year-old female visited Osaka City Sumiyoshi Hospital (Osaka, Japan) for evaluation of a mass in the right side of the vulva. Enucleation of the vulvar tumor was performed and the suspected diagnosis was hemangiopericytoma. The tumor exhibited characteristics of borderline malignancy and the patient was referred to Osaka City University Hospital (Osaka, Japan) for additional therapy. Informed consent was obtained from the patient for the use of clinical information for education and research.

The tissue excised at the previous hospital was histopathologically examined by the pathologist and the suspected diagnosis was PNET. There were no abnormal findings in the physical examination. Magnetic resonance imaging (MRI) and computed tomography did not reveal any residual tumor between the lungs and the vulva.

Simple vulvectomy and resection of the inguinal lymph nodes were performed, showing no microscopic residual tumor presence. The patient underwent outpatient observation.

**Figure 1:**
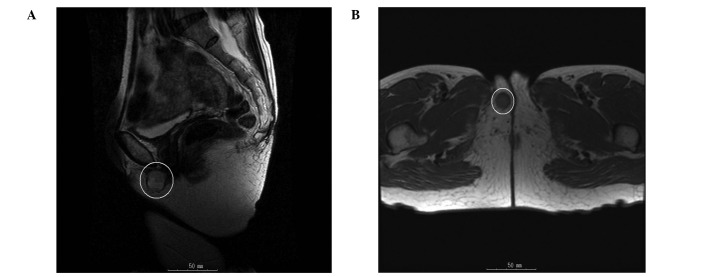
Pelvic magnetic resonance imaging revealed a 3-cm mass in the right side of the vulva. (A) Saggital T2-weighted images demonstrated high signal intensities and (B) Axial T1-weighted images demonstrated low signal intensities.

Four years following the initial surgery, a mass in the right side of the vulva was observed. MRI imaging revealed a 3-cm mass in the right side of the vulva. T1-weighted images demonstrated low signal intensity and T2-weighted images demonstrated high signal intensity (Fig. 1). The possibility of recurrence was suspected and the tumor was excised. The excised tumor was a yellow-white, solid, soft and elastic mass.

### Pathology

**Figure 2:**
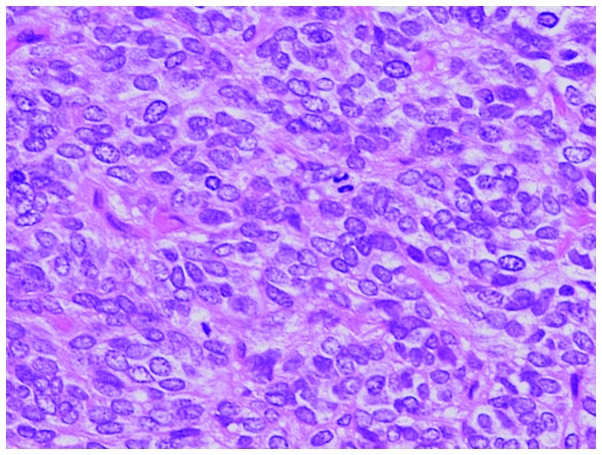
Hematoxylin and eosin-stained sections of the tumor showed a solid growth pattern. The tumor comprised small, round-to-oval nuclei. Homer-Wright rosettes were not observed.

Hematoxylin and eosin-stained sections of the tumor showed a solid growth pattern. The tumor comprised small, round-to-oval nuclei. Homer-Wright rosettes were not observed (Fig. 2).

### Immunohistochemistry

The tumor stained positively for MIC-2, synaptophysin, neuron-specific enolase (NSE) and neurofilament antibodies. The tumor cells were negative for periodic-acid Schiff, vimentin, desmin, chromogranin A, CD34, CD45 [leukocyte common antigen (LCA)], S100, α-smooth muscle actin (α-SMA) and CD117 antibodies (Table I).

### Clinical course

The patient was diagnosed with recurrent PNET that had originated in the vulva. Multidisciplinary therapy was administered. Combination chemotherapy, using vincristine, tetrahydropyranyl-adriamycin and cyclophosphamide (VAC), was administered and radiation therapy, ifosfamide chemotherapy and VAC therapy were subsequently performed. The only adverse event observed with this medical treatment was G3 myelosuppression. Following multidisciplinary therapy, the patient underwent further outpatient observation. To date, the patient remains alive with no evidence of disease four years following the final medical treatment.

## Discussion

PNETs were first reported in 1918 by Stout (2) as small round cell tumors arising in the ulnar bone with rosette formation. Hart and Earle (1) described the term PNET in 1973. PNETs are classified as central or peripheral types based on their site of origin. Peripheral PNETs usually originate in the bone or soft tissue and often from genital organs (3,4). However, a small number of reports have described PNETs originating in the vulva. Fewer than 20 case reports were found in a search of current literature (5–9).

Other small round cell tumor types include neuroblastoma, malignant lymphoma and rhabdomyoblastoma. The tumor in the present case was immunohistologically positive for MIC-2 and, therefore, the patient was diagnosed with an EFT. Notably, markers of nervous system involvement, including NSE, neurofilaments and synaptophysin, were also positive. The patient was therefore diagnosed with recurrent PNET that originated in the vulva. In the present case, neuroblastoma was excluded, as the tumor was immunohistochemically positive for MIC-2, and malignant lymphoma was excluded, as the tumor was immunohistochemically negative for LCA. Rhabdomyoblastoma was also excluded, due to a negative result for desmin and α*-*SMA. However, the immunohistochemistry of PNETs is controversial and identification of the chromosomal translocations is effective in determining the diagnosis. The chimeric chromosomes associated with EWS/PNETs are EWS-friend leukemia virus integration 1 (FLI1) (11;22)(q24;q12), EWS-ETS-related gene (ERG) (21;22)(q22;q12), EWS-ETS variant 1 (7;22)(q22;q12), EWS-EIAF (17;22)(q121;q12) and EWS-FEV (2;22)(q33;q12). All chimeric chromosomes exhibit EWS at the 5′-end and transcription factors of the ETS family, associated with cell proliferation, at the 3′-end. They are classified as EWS/ETS chimeric chromosomes due to this feature. EWS is present on chromosome 22 and gene expression of EWS is observed in various organs. EWS/ETS chimeric chromosomes take part in cell proliferation, through transcription or transformation activities, and affect cultured fibroblasts, thereby promoting neural and epithelial differentiation (10). Overall, >90% of EWS/PNETs contain chimeric chromosomes of EWS-FLI1 and EWS-ERG, in particular EWS-FLI1 translocation, which is present in >80% of EWS/PNETs (11,12).

Poor prognostic factors in EWS/PNETs include lesions of the body trunk, patient ages of ≥15 years, tumor volumes of ≥200 ml, the presence of distant metastasis, high levels of serum lactate dehydrogenase, a poor response to chemotherapy and recurrence within two years. The most unfavorable prognostic factor is the presence of distant metastasis. Even with aggressive treatment, patients with metastases exhibit an ~20% chance of long-term survival (13). Despite having no distant metastatic tumors, the patient in the current case report exhibited unfavorable prognostic factors with regard to the location of the lesion (body trunk) and the age of the individual (60 years).

There are several reports on the management of PNETs. The majority of PNETs are treated as EFTs. Multidisciplinary therapy is often performed for EFTs as recurrence is common. Prior to the advent of modern chemotherapy, <10% of patients with EFT survived beyond five years following diagnosis (14). The Intergroup Ewing’s Sarcoma Study and Cooperative Ewing Sarcoma Study recommend the standard chemotherapy comprising between four and six drugs, including doxorubicin, cyclophosphamide, vincristine, ifosfamide, etoposide and actinomycin (15). However, a standard therapy for recurrent tumors has not been established and autologous peripheral blood stem cell transfusion following chemotherapy is occasionally reported (16). In one study, the five-year survival rate for patients with recurrence at local sites was 31.4±11.6% in the surgical group and 9.1±6.1% in the non-surgical group (17). The effectiveness of the aforementioned chemotherapy or surgery protocols for older patients, as in the present case, remains unclear, due to the majority of studies on the treatment of EFT involving young patients.

The current case report presented a rare case of PNET originating in the vulva. Although PNETs usually exhibit a poor prognosis, the patient remains alive and with no evidence of disease. It is hypothesized that this may be due to the 3-cm tumor size and the absence of distant metastasis at the time of recurrence.

## Figures and Tables

**Table I:** Immunohistochemistry results.

Antibody	Staining result
MIC-2	+
Synaptophysin	+
NSE	+
Neurofilament	+
Chromogranin	−
PAS	−
Vimentin	−
Desmin	−
Chromogranin A	−
CD34	−
CD45	−
S100	−
αSMA	−
CD117	−

NSE, neuron-specific enolase; PAS, periodic-acid Schiff; αSMA, α smooth muscle actin.
